# Integrative Identification of Chloroplast Metabolism-Related RETICULATA-RELATED Genes in Soybean

**DOI:** 10.3390/plants14101516

**Published:** 2025-05-19

**Authors:** Qianli Dong, Lu Niu, Xiyu Gong, Qianlong Xing, Jie Liang, Jun Lang, Tianya Wang, Xiangdong Yang

**Affiliations:** 1 Key Laboratory of Molecular Epigenetics of Ministry of Education (MOE), Northeast Normal University, Changchun 130024, China; gongxiyu110@nenu.edu.cn (X.G.); xingql983@nenu.edu.cn (Q.X.); liangjie109@nenu.edu.cn (J.L.); langj@nenu.edu.cn (J.L.); 2 Jilin Provincial Key Laboratory of Agricultural Biotechnology, Jilin Academy of Agricultural Sciences, Changchun 130033, China; niulu@jaas.com.cn

**Keywords:** soybean, RETICULATA-RELATED genes, chloroplast development, RNA/protein structural analysis, transcriptomics analysis

## Abstract

As a globally important leguminous crop, soybean (*Glycine max* L.) serves as a vital source of edible oils and proteins for humans and livestock. Oils in leaves can help crops combat fungal infections, adapt to temperature changes via fatty acid modulation, and support resource recycling during leaf senescence. However, accumulating oils in leaves is a fundamental challenge due to the need to balance the inherently competing photosynthesis and fatty acid biosynthesis processes within chloroplasts. RETICULATA-RELATED (RER), known to regulate chloroplast function and plastid metabolism in Arabidopsis, plays an essential role in leaf development. Here, 14 non-redundant *GmRER* genes were identified in soybean and phylogenetically classified into four subclades. Most Arabidopsis *RER* genes were evolutionarily preserved as gene duplicates in soybean, except for *GmRER5* and *GmRER6*. RNA secondary structures spanning the coding sequences (CDSs), the 5′- and 3′- untranslated regions (UTRs) of *GmRERs*, displayed exceptional structural plasticity in CDSs, while exhibiting limited conservation in UTRs. In contrast, protein structures retained conserved folds, underscoring evolutionary constraints on functional domains despite transcriptional plasticity. Notably, GmRER4a and GmRER4b represented an exceptional case of high similarity in both protein and RNA structures. Expression profiling across fourteen tissues and three abiotic stress conditions revealed a dynamic shift in expression levels between leaf-predominant and root-enriched *GmRER* paralogs after stress treatments. A comparative transcriptome analysis of six soybean landraces further revealed transcriptional polymorphism in the *GmRER* family, which was associated with the expression patterns of lipid biosynthesis regulators. Our comprehensive characterization of GmRERs may offer potential targets for soybean breeding optimization in overall plant oil production.

## 1. Introduction

Soybean (*Glycine max* L.), a globally vital legume crop, serves as a primary source of plant-derived protein and oil [1,2,3,4]. By now, advances in scientific technologies have enabled the identification of a growing number of genes associated with soybean seed oil content, facilitating improvements in both oil yield and quality [5,6,7,8,9,10,11]. Although the global demand for vegetable oils from plants is continuously rising, the cultivation areas and productivity of conventional oil crops remain limited. Therefore, innovative strategies to produce oils from non-seed biomass, with a particular focus on leaf tissues, have been developed [12,13,14,15]. Previous studies have demonstrated that oils in soybean leaves fulfill multiple physiological roles. First, they function as subcellular factories producing antifungal compounds such as oxylipins, which protect against pathogens and support seedling survival [16,17,18]. Second, during leaf senescence, oil accumulation provides transient lipid reserves and precursor molecules critical for nutrient remobilization [16,18,19]. Additionally, leaf oils store bioactive secondary metabolites that aid in stress adaptation and defense mechanisms [17,19]. The fatty acid composition of leaf oil dynamically adjusts to environmental conditions; for instance, elevated levels of polyunsaturated fatty acids (e.g., linolenic acid) at high temperatures enhance thermotolerance [16].

As chloroplasts are central to fatty acid biosynthesis, photosynthesis, and stress signaling [20,21], understanding and exploring the regulatory mechanisms of chloroplast development and identifying key regulatory genes for leaf oil production in soybean are of great importance. The RETICULATA-RELATED (RER) gene family has emerged as a key player in chloroplast maintenance and plastid metabolism [22,23,24]. Over the past few decades, research endeavors have extensively documented the Arabidopsis RETICULATA-RELATED gene family, which consists of six genes encoding proteins with high sequence similarity to *RE* [22,25,26]. These genes, named *RER1* to *RER6*, are plant-specific and found in most sequenced plant genomes. The RER proteins can be categorized into three pairs (RE-RER1, RER2-RER3, and RER5-RER6) and one single protein, RER4, based on gene structure and amino acid sequence similarity. Most of the RER family proteins contain a conserved plant-specific domain, DUF3411, while some, like RER2, RER3, and RER4, also contain additional transmembrane helices, like TM3, consistent with their membrane localization [22]. RER5 and RER6 retain the conserved DUF3411 domain but are distinguished by an additional C-terminal DUF399 domain, a feature correlating with their localization in the thylakoid lumen [27,28]. The RER family can be divided into four functional modules with distinct roles [22]. For instance, RE and RER3 have redundant roles in downstream biosynthetic pathways of pyruvate, which are crucial for the generation of acetyl-CoA and de novo fatty acid synthesis in leaves; RER3 plays a specific role in embryogenesis in response to auxin; and RER5 and RER6 show functional redundancy in constituting the essential components of thylakoids [22,29].

Gene family expansion through duplication is a hallmark of plant genome evolution, enabling functional diversification and adaptation to environmental challenges [30,31,32]. Moreover, the interplay between genetic redundancy, regulatory plasticity, and protein structural conservation in polyploids remains poorly resolved [30,33]. Soybean, as a diploidized tetraploid, offers a unique system to study the lineage-specific expansions and subfunctionalization of family genes [33,34]. As described above, AtRER members are known to regulate chloroplast retrograde signaling and stress responses [22,23], the mechanisms by which duplicated RER paralogs partition roles across tissues and stresses are unclear. Thus, the evolutionary history, structural diversity, and functional roles of RER homologs in soybean, a paleopolyploid species with a complex genome shaped by whole-genome duplication events [34,35], need to be illustrated.

Here, we conducted genome-wide identification, structural characterization, and a comparative transcriptome analysis of the GmRER family in soybean. We investigated the evolutionary dynamics of GmRERs diversification, dissected their regulatory architectures, unraveled their spatiotemporal roles in stress adaptation, and displayed their natural transcriptional plasticity in six soybean landraces. Our study revealed how soybean paleopolyploid history sculpted the GmRER family into functionally compartmentalized subclades, balancing conserved chloroplast metabolic roles with lineage-specific innovations in transcriptional regulation. The findings from our study provide comprehensive and systematic information for further studies of the effects of the GmRER gene family on soybean leaf development regulation, and shed light on dissecting the molecular mechanism of GmRER proteins; hence, they may contribute to the leaf oil breeding of soybean in the future.

## 2. Results

### 2.1. Identification of the GmRER Gene Family

To identify members of the RETICULATA-RELATED (RER) gene family in soybean (*Glycine max*), we first retrieved the protein sequences of Arabidopsis RE and six RER genes (AtRER1 to AtRER6) from TAIR (https://www.arabidopsis.org/ (accessed on 1 April 2025)), including their conserved DUF3411 domain (PFAM family PF11891). These sequences served as queries for bidirectional BLASTP searches (e-value ≤ 1 × 10^−10^) against the soybean proteome (assembly Wm82.a4.v1 from Phytozome v13) [36], followed by HMMER validation using the PF11891 profile (cutoff: 1 × 10^−50^) to ensure domain integrity. Redundant sequences were removed by manual curation and InterProScan confirmation. In total, 14 non-redundant *GmRER* genes were identified (Table S1 and Table 1). Notably, duplication type analysis revealed that most GmRER paralogs originated from whole-genome duplication (WGD), whereas GmRER4c was derived from dispersed duplication, suggesting distinct evolutionary origins within the GmRER4 subfamily.

**Figure 1:**
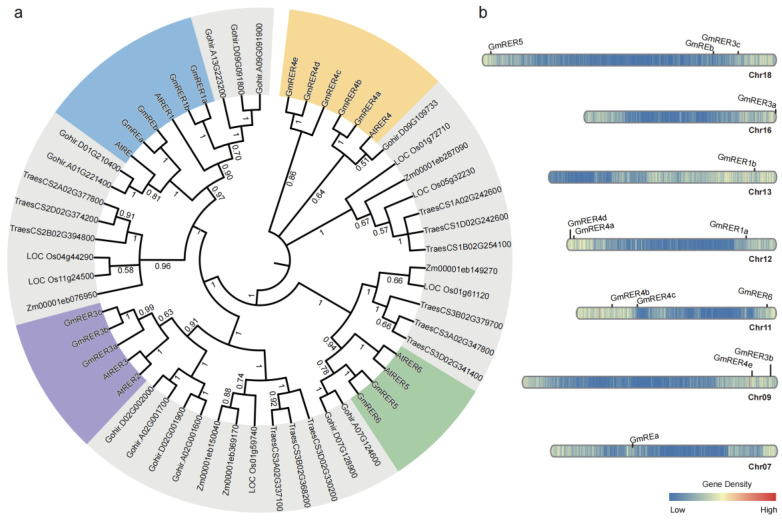
Phylogenetic analysis and genomic distribution of RER family genes in soybean and related species. (**a**) Rooted maximum-likelihood (ML) phylogenetic tree of RER proteins from six species: *Oryza sativa* (rice), *Gossypium hirsutum* (cotton), *Zea mays* (maize), *Triticum aestivum* (wheat), *Arabidopsis thaliana* (Arabidopsis), and *Glycine max* (soybean). Numbers at nodes indicate percentage of replicate trees (500 replicates) in which associated taxa are clustered together, representing branch support confidence. Soybean names are labeled as *GmRERs* and Arabidopsis names as *AtRERs*, while identifiers (IDs) from rice, cotton, maize, and wheat are retained as annotated in their respective genomes. Four major subclades containing GmRERs and AtRERs were represented in colors. (**b**) Chromosomal distribution and regional duplication of 14 *GmRER* genes in *Glycine max*. Locations of 14 *GmRER* genes on soybean chromosomes (07, 09, 11, 12, 13, 16, 18) are shown according to Wm82.a4.v1 of soybean genome annotation. Color bar represents gene density distribution on chromosomes.

The phylogenetic architecture of soybean GmRERs was reconstructed through a comparative analysis of RER homologs from six species (*Arabidopsis thaliana*, *Glycine max*, *Oryza sativa*, *Zea mays*, *Gossypium hirsutum*, and *Triticum aestivum*). The resulting phylogeny delineated clear monocot–dicot divergence, with homologs from rice, maize, and wheat forming clades, distinct from those of Arabidopsis, soybean, and cotton (Figure 1a). Four major subclades were identified for the 14 soybean GmRER proteins (RE/RER1, RER2/RER3, RER4, and RER5/RER6). The Arabidopsis single-copy orthologs AtRE and AtRER1 corresponded to duplicated soybean GmREa/b and GmRER1a/b paralogs, suggesting post-speciation duplication events in soybean. A notable expansion occurred in soybean RER4 lineage, generating five soybean paralogs, GmRER4a–e. Intriguingly, three GmRER paralogs co-clustered with AtRER2 and AtRER3 in one subclade. Through reciprocal BLASTP analysis, the homology with AtRER2 was excluded, warranting their designation as GmRER3a–c. Although GmRER5 and GmRER6 were phylogenetically aligned together with AtRER5 and AtRER6, bidirectional best-hit BLASTP analysis specified their relationships, with GmRER5 best matching AtRER5, and GmRER6 best matching AtRER6.

Chromosomal localization indicated that *GmRER* genes were localized across seven chromosomes (Chr07, 09, 11, 12, 13, 16, 18), revealing duplication patterns tied to polyploidization (Figure 1b). Tandem duplications on chromosomes 09, 16, and 18 generated the *GmRER3* cluster (*GmRER3a–c*), while *GmRER4* paralogs (*GmRER4a–e*) were scattered on chromosomes 09, 11, and 12 via segmental duplications. Intriguingly, *GmRER5/6*, positioned as sister clades on chromosomes 11 and 18, mirrored the Arabidopsis *AtRER5/AtRER6* chromosome location (Chr.2/Chr.3). In general, the *GmRER* genes were unevenly distributed on chromosomes 07, 09, 13, and 16, but were relatively evenly distributed near the telomeres of chromosomes 11, 12, and 18 (Figure 1b).

### 2.2. Architectural Diversity of GmRERs in Gene Structures and Conserved Motifs

**Figure 2:**
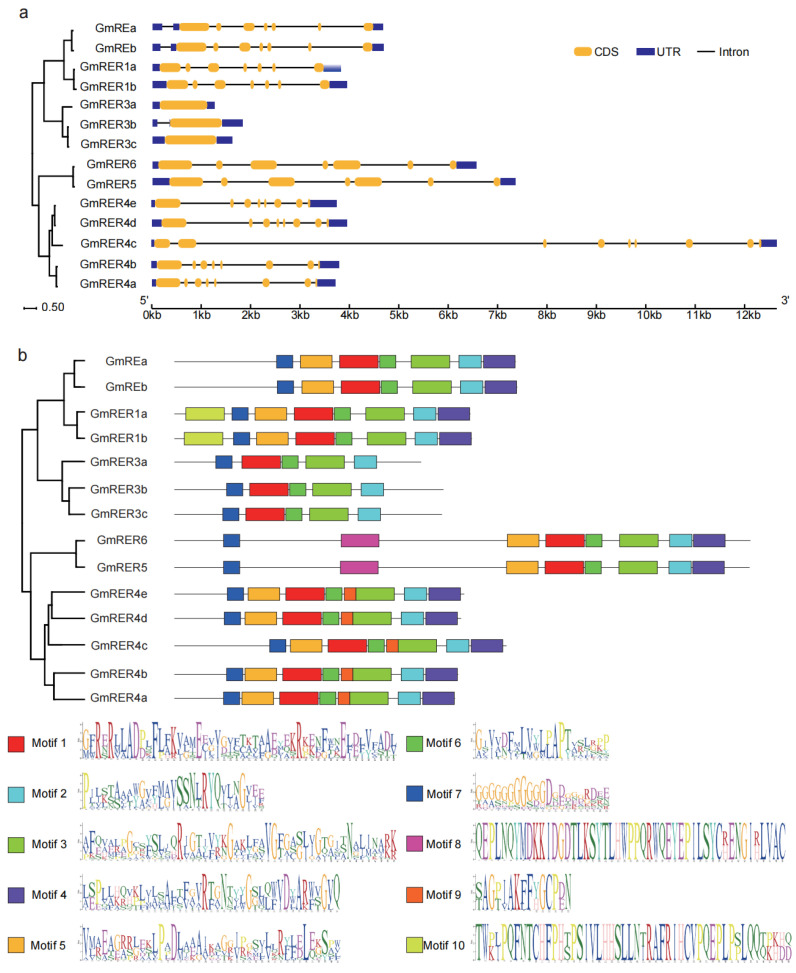
Gene structure and motif distribution of GmRERs. (**a**) The gene structures of *GmRER*s were described using the GSDS tool. The yellow rectangles represent the coding sequences (CDS), the blue rectangles indicate the untranslated regions (UTRs), and the straight lines denote the introns. (**b**) The motif distributions within GmRER proteins were identified using the MEME tool. The GmRER proteins are drawn to scale. The position of each colored block indicates the location of a motif with a matching sequence.

The structural plasticity and functional diversification of GmRERs were unveiled by an in-depth analysis of exon–intron architectures and conserved motifs. As shown in Figure 2a, the *GmRERs* exhibited subclade-specific structural patterns. Notably, *GmRER3* paralogs exhibited divergent exon-intron architectures. *GmRER3a* and *GmRER3c* were intronless, in accordance with their Arabidopsis ortholog *AtRER3* [22], while *GmRER3b* retained a single intron. Members of the *GmRER1* and *GmRER5/6* subclades retained conserved seven-exon structures, resembling their Arabidopsis counterparts [22], whereas the expanded *GmRER4* subclade diverged (Figure 2a). *GmRER4a/b/d/e* retained eight exons, but *GmRER4c* acquired a ninth exon via intron retention in the 3′ UTR, which may potentially influence transcript stability or alternative splicing. This gene structural divergence of *GmRERs* suggests an evolutionary interplay between genetic drift and selection during soybean domestication.

Based on MEME-based motif profiling, 10 conserved motifs (five universal, five subclade-specific) were identified (Figure 2b, Table S2). GmRER paralogs within a subclade shared similar motif content, which was the case for their phylogenetic relationships (Figure 1a). The distinct motif organization patterns across subclades of GmRERs suggested their probable functional divergence. Motifs 1–3 and 6–7 were universal, suggesting that their encoding domains are essential for functions of GmRERs (Figure 2b). Intriguingly, while GmRER3 paralogs formed a tightly clustered clade in the phylogenetic tree, they uniquely lacked motif 5, which was otherwise conserved in other subclades. Coupled with the intronless configuration of *GmRER3a* and *GmRER3c* in gene structures (Figure 2a), this implied that GmRER3 paralogs might have distinct regulatory strategies in transcription. Furthermore, GmRER4 paralogs uniquely retained motif 9, which was predicted to be an MYB-binding site, indicating they could be potential targets for MYB transcription factors.

### 2.3. RNA Secondary Structure and Protein Structure Prediction of GmRERs

**Figure 3:**
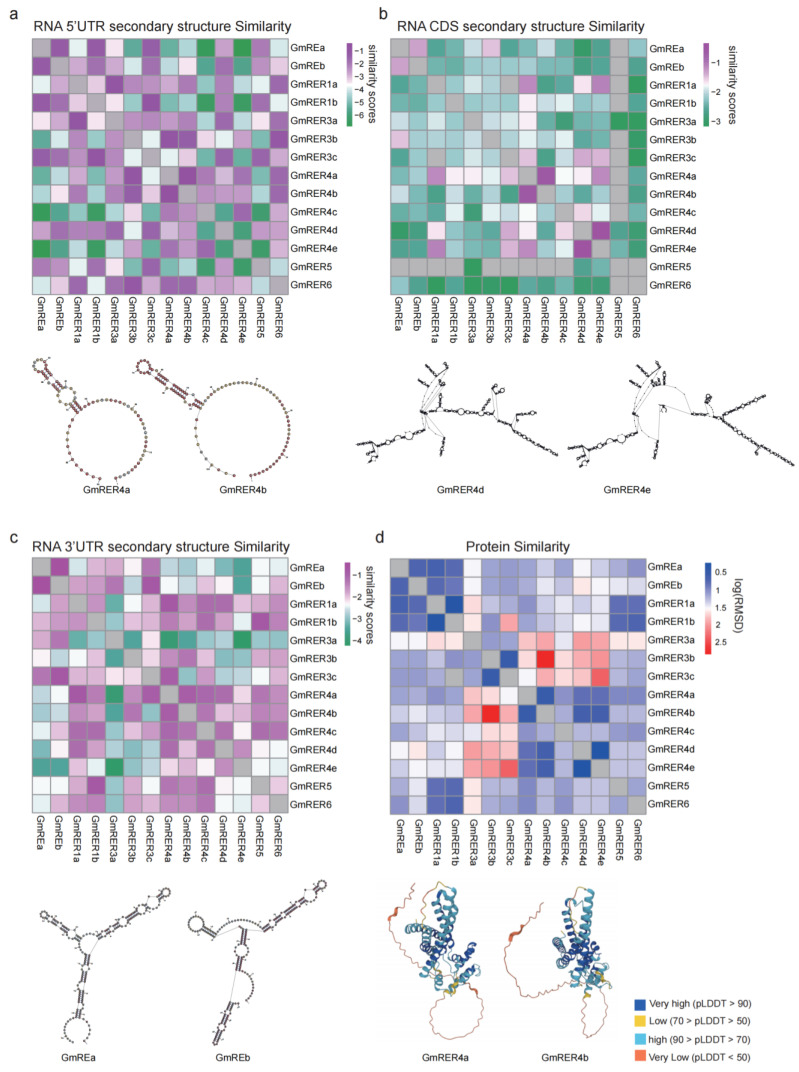
The structural similarity of GmRER mRNA and proteins. (**a**–**c**) The RNA secondary structure similarity of GmRER mRNA in the 5′ UTR (**a**), CDS (**b**), and 3′ UTR (**c**) regions. The values represent similarity scores calculated by RNAForester, with higher scores indicating greater similarity (purple) and lower scores indicating lower similarity (green). (**d**) The protein structure similarity of GmRERs, represented by log(RMSD) values calculated using PyMOL. Lower log(RMSD) values indicate higher similarity (blue), while higher values indicate lower similarity (red). Structure similarities which cannot be calculated are labeled in gray, and below the heatmaps are examples of some of the most conserved structure pairs.

With the purpose of understanding the potential mechanisms of GmRERs in post-transcriptional regulation and functional performance, we analyzed their RNA secondary structures and 3D protein structures. To increase the reliability of the prediction outcomes, the RNA secondary structures of 5′- and 3′- UTRs and CDS regions were analyzed separately (Figure S1). Then, we quantified the similarity of the RNA structures using RNAforester-based comparisons. The heatmaps in Figure 3a–c indicate that the RNA secondary structures of *GmRER* paralogs were remarkably different from each other. Among these structures, 5′ and 3′ UTRs were slightly conserved across the *GmRER* family genes (Figure 3a,c), while CDS regions displayed distinct paralog-specific structural divergence (Figure 3b), suggesting selective pressure on post-transcriptional regulatory mechanisms. Specifically, *GmRER4* paralogs formed a clustered group with homologous RNA architectures (CDS region); *GmRER3* paralogs, despite lacking introns, exhibited exceptional CDS structural plasticity, implying evolutionary dynamics independent of coding sequence conservation (Figure 3b).

Protein structures of GmRERs from the AlphaFold protein structure database were obtained (Figure S2), and the local accuracy of most of them had high confidence values (pLDDT value > 70, colored in blue). Also, we calculated the log(RMSD) values to compare the similarity of protein structures using PyMOL. Unlike the RNA secondary structures, the protein structures were highly conserved across GmRER paralogs (Figure 3d and Figure S2). For example, GmRER1 paralogs retained conserved α-helical topologies, despite lineage-specific variations in RNA structural organization. Likewise, GmRER4 paralogs with highly divergent RNA structures (CDS regions) maintained nearly identical β-sheet-dominated folds in their DUF3411 functional domains. Notably, GmRER4a and GmRER4b shared remarkably similar protein structures and RNA structures (Figure 3a,d), suggesting strong functional constraints across GmRER4 paralogs.

### 2.4. Modular Functionality of GmRER Paralogs via Subcellular Specialization and Lineage-Specific PPI Networks

**Figure 4:**
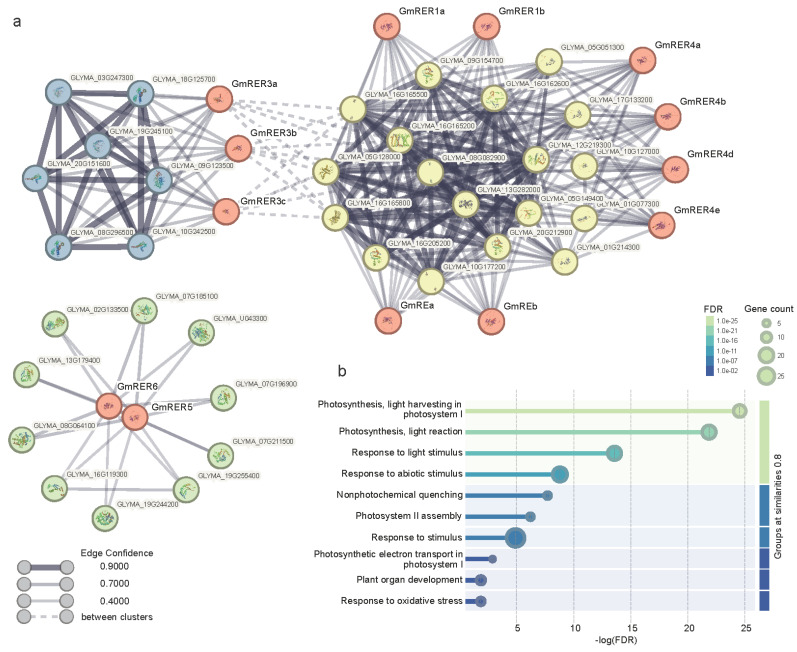
Protein–protein interactions of GmRERs and GO analysis of interacting proteins. (**a**) Protein–protein interaction networks of GmRERs were constructed based on STRING predictions. The nodes of GmRER proteins are labeled in red, and the nodes of other interacting proteins are labeled in yellow, blue, and green, according to the clusters they belong to. Proteins with known or predicted structures are specified in the nodes. Edge width and color represent the scores for edge confidence. (**b**) The lollipop plot displays the GO analysis results of GmRER interacting proteins. The x-axis represents the −log(FDR) values, showing the significance level, and the y-axis shows the significantly enriched GO terms.

For further information on the principal function for GmRER proteins, the physicochemical properties and predicted subcellular localization of GmRERs are summarized in Table 1. Consistent with the phylogenetic relationships, most of the GmRER paralogs shared similar protein pI (isoelectric point), protein MW (molecular weight) and chloroplast localization, except for GmRER5/6. Then, we conducted a STRING-based protein–protein interaction (PPI) analysis to understand the role of GmRERs in the regulatory network of soybean growth and development (Table S3). As shown in Figure 4a, the PPIs of GmRERs can be divided into three clusters. Cluster I consisted of GmRE paralogs, GmRER1 paralogs, GmRER4 paralogs, and their interacting proteins. Cluster II was composed of GmRER3 paralogs and their interacting proteins. Clusters I and II shared some weak interactions with each other. However, the third cluster had no interaction with other GmRERs; it was formed of GmRER5 and GmRER6 along with their interacting proteins.

Gene Ontology (GO) analysis of all of the interacting proteins illustrated that the top three significantly enriched GO terms were “photosynthesis, light harvesting in photosystem I”, “Photosynthesis, light reaction”, and “Response to light stimulus” (Figure 4b), which were in line with the previously reported function of RERs in Arabidopsis [22]. Besides photosynthesis-related GO terms, we found that GmRERs can also interact with proteins functionally enriched in “Response to stimulus”, “Nonphytochemical quenching”, “Plant organ development”, and “Response to oxidative stress” (Figure 4b), which corroborated the well-documented regulatory roles of RERs in *Arabidopsis thaliana* [20,22,24].

Subcellular localizations of GmRER paralogs can explain their functional modules properly (Table 1 and Table S3, Figure 4). Specifically, chloroplast-localized GmRE/RER1/RER4 paralogs interacted directly with photosynthesis-related proteins (such as Glyma.08G082900, Glyma.05G128000, and Glyma.16G165500). Notably, GmRER3 targeted chloroplasts and also interacted with transcriptional machinery proteins (such as Glyma.18G125700, Glyma.19G245100, and Glyma.10G242500), which may bridge the chloroplast–nuclear regulation critical for coordinating transcription in photosynthetic processes. However, plasma membrane-localized GmRER5/6 interacted with redox networks proteins (Glyma.07G185100, Glyma.19G244200, and Glyma.19G255400) separately, predicting their roles in oxidative stress mitigation.

### 2.5. Spatiotemporal Expression Dynamics Define Functional Classes of GmRERs

**Figure 5:**
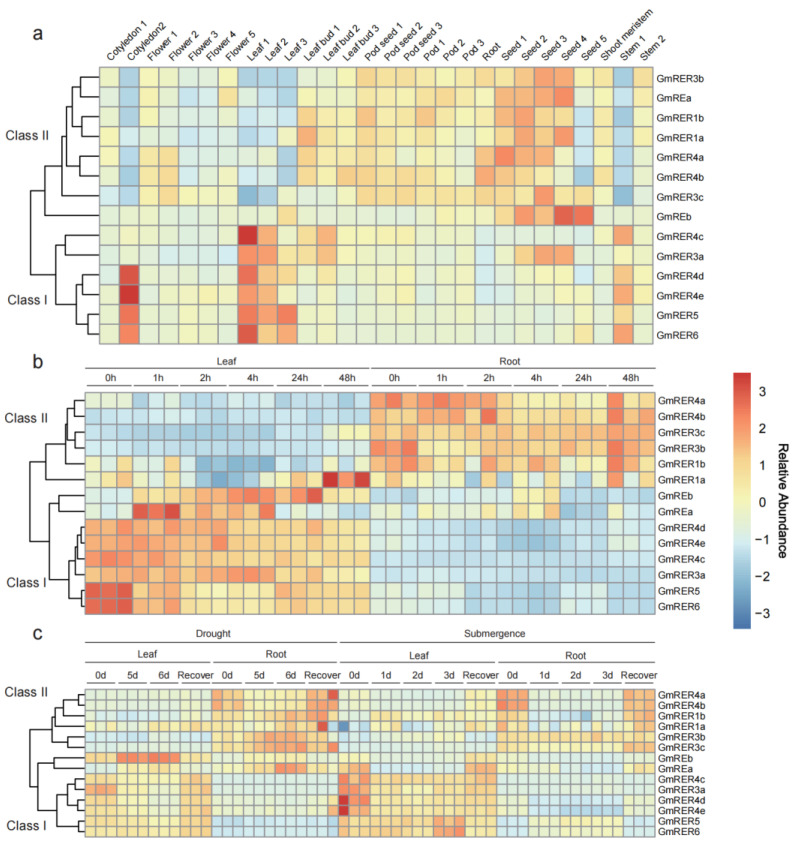
Expression patterns of *GmRERs* in various tissues, developmental stages, and stress conditions. (**a**) Expression of *GmRERs* in cotyledons, roots, stems, leaves, flowers, pods, and seeds at different developmental stages (PRJNA238493). (**b**) Expression of *GmRERs* in leaves and roots under salt stress at six time points (0, 1, 2, 4, 24, and 48 h) with three biological replicates (PRJNA246058). (**c**) Expression of *GmRERs* in leaves and roots under drought and submergence stress at multiple time points (drought stress: 0d, 5d, 6d, recovery; submergence stress: 0d, 1d, 2d, 3d, recovery) with three biological replicates (PRJNA574626). In heatmap, red indicates high expression, and blue indicates low expression.

Chloroplasts, integral to numerous critical metabolic processes in plants [37,38], enable plants to adapt to environmental changes via retrograde signaling pathways between plastids and the nucleus [39,40,41]. Thus, we compared the expression profiles of the *GmRERs* across diverse tissues in various developmental stages and stress conditions, using three published transcriptome datasets from previous studies [42,43,44]. The expression patterns of the *GmRER* paralogs across diverse tissues and stress conditions delineated the functional dichotomy, which can be categorized into two distinct classes with opposing regulatory strategies (Figure 5). Class I genes, comprising *GmRER3a*, *GmRER4c-e*, *GmRER5*, and *GmRER6*, were predominantly expressed in photosynthetic tissues, such as young leaves and cotyledons (Figure 5a). In Figure 5b, after salt treatment, the expression levels of Class I members were rapidly down-regulated in soybean leaves, reaching minimal expression levels within 4 h. While in roots, Class I members maintained basal-level expression under salt stress. Interestingly, *GmREa/b* were clustered into the Class I expression pattern under salt stress, and they ectopically activated leaf-silencing expression (Figure 5a,b). In contrast with salt stress, drought elicited a delayed response from Class I members in leaves, with transcripts sharply declining after 5 days but surging during recovery (Figure 5c). Moreover, submergence stress uniquely induced sustained up-regulation of *GmRER5/6* in leaves and ectopic activation of *GmRER5/6* in roots (Figure 5c).

Class II genes, including *GmREa/b, GmRER1a/b*, *GmRER3b/c*, and *GmRER4a/b*, displayed inverse tissue expression patterns compared with those of Class I genes, and were constitutively expressed in seeds, pods, and roots (Figure 5a). According to Figure 5b,c, the expression of Class II genes was stress-induced. Salt stress moderately up-regulated the expression of *GmRER4b* in roots at 48 h, while drought stress triggered the root-specific induction of *GmRER3b/c* expression. Submergence disrupted the tissue-specific expression of *GmRER1a/b* and induced its ectopic activation in leaves (Figure 5c). In summary, the stress-induced reciprocal expression shifts between leaf-predominant and root-enriched *GmRER* paralogs underscore their opposing regulatory strategies for photosynthesis and energy metabolism.

### 2.6. Cis-Regulatory Modules Conveyed Roles of GmRERs in Stress and Development

**Figure 6:**
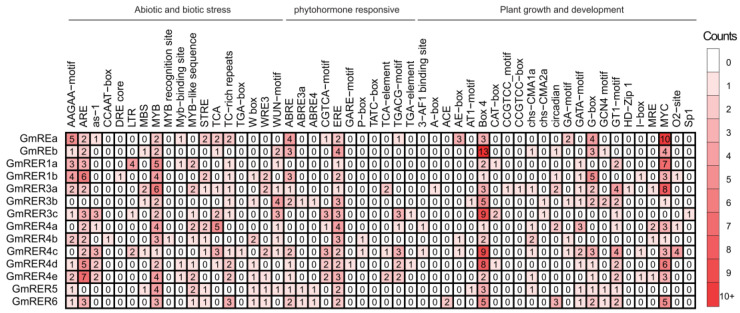
Statistical analysis of *Cis*-acting elements in the promoter regions of *GmRERs*. *Cis*-elements are categorized into three main classes: abiotic and biotic stress, phytohormone-responsiveness, and plant growth and development. The numbers represent the occurrence of each *cis*-element in the promoter regions of the genes. The deeper the red color, the higher the frequency of occurrence.

As is well known, the expression patterns of genes are controlled by their promoters [45]. Thus, we performed an analysis of 2 kb promoter regions across *GmRER* family genes, and 52 categorized *cis*-regulatory elements were identified (Figure 6). Based on their previously reported functional annotations, these *cis*-elements were partitioned into three major classes, including plant growth/development (23 categories, 44.2%), abiotic/biotic stress (18 categories, 34.6%), and phytohormone-responsiveness (11 categories, 21.1%).

Combined with our expression data (Figure 5), stress-responsive *cis*-regulatory motifs were found to be enriched in *GmRERs* with tissue-specific expression (Figure 6). For instance, root-expressed *GmRER1b* harbored three ABREs (abscisic acid-responsive elements), which was reported to be related to drought induction [46], while predominantly leaf-expressed *GmRER5/6* uniquely combined two ethylene-responsive elements (EREs) with four TC-rich repeats, which was proven to be linked to salt stress adaptation [47]. For phytohormone-responsive modules, *GmRER3a* and *GmRER3b* carried both ABA-responsive ABREs (one–two motifs) and SA-responsive TCA elements (one motif each), while *GmRER3c* retained only SA-responsive TCA motifs (two motifs), enabling partitioned drought–pathogen defense strategies [48]. In contrast, GmRER5/6 promoters combined ethylene-responsive elements (EREs; two motifs per promoter) with photoperiod-associated circadian motifs, suggesting that they might both involved in light-entrained ethylene signaling during hypoxia recovery [49]. Moreover, development-related motifs diverged sharply. Specifically, *GmRER4c/d* contained eight–nine Box-4 elements (light-responsive) and one–three G-box motifs (light-responsive), and *GmRER3a* retained one HD-Zip-I motif, which may potentially be linked to chloroplast differentiation [50]. Additionally, although *GmREa* retained 10 MYC motifs, 4 G-box motifs (light responsiveness), and 2 MYB sites, barely any obvious expression changes in *GmREa* were found under the tested conditions in our expression data (Figure 5), suggesting that transcriptional regulatory suppression may occur despite its functional *cis*-element repertoire.

### 2.7. Expression Patterns of GmRERs in Six Northern Spring Soybean Landraces

**Figure 7:**
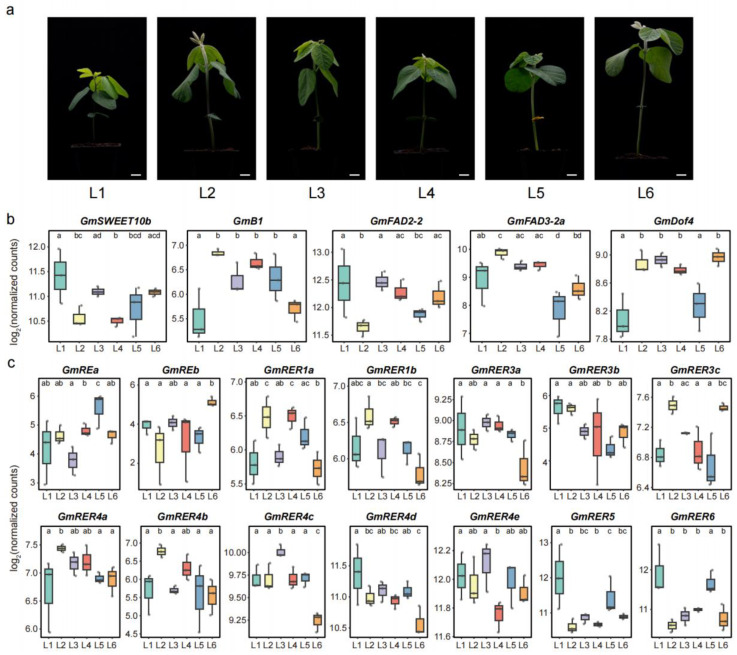
Expression levels of *GmRER* family genes in six northern spring soybean landraces and their seedling phenotypes. (**a**) The seedling phenotypes of the six landraces at the soybean V1 stage. The representative images of accessions labeled L1 to L6 correspond to the following landraces (left to right): Xiaoli Moshidou (L1), Baimaoshuang (L2), Tiejia Silihuang (L3), Heidadou (L4), Zhouye (L5), and Aqi Manjinhuang (L6). Scale bars = 1.2 cm. (**b**) The expression patterns of five known oil biosynthesis-associated genes (*GmSWEET10b*, *GmB1*, *GmFAD2-2*, *GmFAD3-2a*, and *GmDof4*) across the six landraces. The data represent Log2-transformed normalized counts derived from DESeq2. (**c**) Differential expression analysis of GmRER family genes in the six landraces. For panels (**b**,**c**), statistical significance was determined by pairwise comparisons using DESeq2’s Wald test. Lowercase letters above the boxplots indicate significant differences: landraces sharing the same letter are not significantly different, while distinct letters denote statistically divergent expression (*p* < 0.05).

Previous studies have demonstrated that genetic diversity bottlenecks occurred during soybean domestication and subsequent improvement [51,52]. Comprehensive identification and characterization of natural genetic variations are therefore essential for advancing soybean breeding programs [53,54,55,56]. To investigate expression variability within the *GmRER* gene family across natural collections, we conducted comparative transcriptome analyses of six northern spring soybean landraces, including Xiaoli Moshidou (L1), Baimaoshuang (L2), Tiejia Silihuang (L3), Heidadou (L4), Zhouye (L5), and Aqi Manjinhuang (L6). These accessions exhibited pronounced phenotypic variation in leaf growth and development at the vegetative stage 1 (V1 stage; Figure 7a).

We generated six RNA-seq datasets from the first trifoliate leaves of the six landraces, which were aligned to the cultivated soybean reference genome (*Glycine max* Williams 82, assembly version a4v1, Table S4). Prior to cross-landrace comparisons, the RNA-seq data quality was validated through Spearman’s correlation analysis, demonstrating high inter-replicate concordance (*R* > 0.95) across three biological replicates per accession (Figure S3). First, we analyzed the expression levels of five well-known genes involved in lipid biosynthesis, including *GmSWEET10b* [57], *GmB1* [58], *GmFAD2-2* [5], *GmFAD3-2a* [6], and *GmDof4* [7]. These five genes exhibited distinct expression patterns among the six landraces. Among them, three genes (*GmSWEET10b*, *GmFAD2-2*, and *GmFAD3-2a*) showed relatively more frequent significant differences in the pairwise landrace comparisons (Figure 7b). These findings indicate expression polymorphism in key regulatory genes associated with soybean oil biosynthesis among the studied landraces.

Then, we evaluated the expression variations in *GmRER* genes among the six landrace accessions. As shown in Figure 7c, most *GmRER* genes predominantly displayed landrace-specific expression divergence. Within the *GmRER3* group, *GmRER3a* and *GmRER3b* displayed limited expression variation, whereas *GmRER3c* exhibited statistically significant differences in expression across landraces. Similar divergence patterns were observed for *GmRE* paralogs and *GmRER4* paralogs, with distinct expression trajectories among landraces (Figure 7c). Strikingly, *GmRER1* paralogs (GmRER1a/b) both showed prominent expression divergence and highly similar expression patterns among the six landrace accessions. In addition, pairwise comparisons revealed that paralog groups diverged more substantially in their expression profiles than *GmRER* members within the groups. Thus, the expression variation dynamics within and between paralog groups differed substantially among GmRER family members across landrace accessions.

## 3. Discussion

### 3.1. Evolutionary Conservation and Functional Dynamics in the GmRER Family

The RER gene family has been evolutionarily conserved across land plants to regulate chloroplast development and lipid metabolism [22,25,26], yet lineage-specific expansions in crops like soybean have sculpted unique functional configurations (Figure 1a) [30,35]. Our genome-wide characterization revealed fundamental divergences between soybean and Arabidopsis in the RER family composition. Most strikingly, while Arabidopsis harbors both RER2 and RER3 clades [22], soybean exclusively retains three GmRER3 paralogs clustered with the Arabidopsis RER2-RER3 branch (Figure 1a). Reciprocal BLASTP analysis excluded the homology to AtRER2, indicating either functional divergence or loss of GmRER2 paralogs in soybean. This absence may reflect selective pressure favoring GmRER3 paralogs optimized for legume-specific environmental adaptations. Notably, the intronless configuration of *GmRER3a/c* (versus single-exon *AtRER3*; Figure 2a), facilitates rapid transcriptional responses to drought (Figure 5c), aligning with soybean susceptibility to water deficits during vegetative growth [16,59].

In contrast to Arabidopsis’ single *RER4* copy [22], soybean has retained five *GmRER4* paralogs via paleopolyploidy-driven segmental duplications (Figure 1). This expansion likely evolved to support soybean heightened demand for lipid-mediated stress adaptation and resource allocation in leaves [16,18]. Structural conservation in core DUF3411 domains (Figure 3d), alongside promoter diversification (MYB motif enrichment; Figure 6), enables *GmRER4* paralogs to balance constitutive roles in vascular nutrient transport (Figure 5a) with stress-responsive transcription plasticity (Figure 4b). Such subfunctionalization aligns with the dosage balance hypothesis [60], where polyploid/paleopolyploid species preferentially retain duplicated metabolic regulators to maintain stoichiometric relationships in specialized pathways, like nitrogen fixation or lipid reserves during senescence [61,62].

These evolutionary trajectories have practical implications for soybean breeding. The drought-inducible *GmRER3a/c* and hypoxia-responsive *GmRER5/6* identified here (Figure 5) offer candidate loci for breeding climate-resilient soybeans. However, functional redundancy among paralogs poses challenges for gene editing, in that knocking out single *GmRER4* members (e.g., GmRER4d) may not yield phenotypic changes unless multiple paralogs are targeted [63]. Future studies should employ multiplex CRISPR systems to dissect additive effects and prioritize hub paralogs (e.g., high-expression *GmRER4e*) for metabolic engineering.

### 3.2. Structural Diversification Coupled with Regulatory Plasticity in GmRERs

Take *GmRERs* as an example. Soybean has fostered structural innovations that decouple RNA-level plasticity from protein functional conservation (Figure 3). Meanwhile, RNA secondary structures of *GmRER3* and *GmRER4* paralogs diverged markedly in their CDS regions (Figure 3b), while their protein core folds remained conserved (Figure 3d). The preservation of core protein structures despite RNA heterogeneity likely buffered soybean against genomic perturbations while permitting regulatory diversification. For example, *GmRER4* paralogs acquired MYB-responsive promoters (Figure 6) without disrupting DUF3411 domain integrity, enabling tissue-specific lipid mobilization during leaf senescence, which may serve as a critical adaptation for nutrient recovery in soybean’s determinate growth habit [16,18,62].

Divergent subcellular localization further partitions paralog functions. Unlike chloroplast-anchored GmRER1/RER4 paralogs, *GmRER5/RER6* targeted the plasma membrane (Table 1) and were involved in redox regulation through interactions with NADP-oxidoreductases (Figure 4). This subcellular rewiring parallels their ethylene-responsive promoter motifs (Figure 6), suggesting neofunctionalization to mitigate hypoxia-triggered oxidative stress in soybean roots, a rare but devastating challenge in submerged fields [24,64]. Such compartment-specific adaptations highlight how structural diversification enables paralogs to resolve conflicting evolutionary pressures (e.g., stress tolerance vs. developmental growth).

### 3.3. Functional Implications of GmRERs in Leaf Lipid Metabolism

Our results unveil soybean’s hierarchical resource allocation strategy under stress, mediated by the spatiotemporal polarization of *GmRER* paralogs (Figure 5), Class I genes (mainly expressed in photosynthetic tissues; Figure 5a) are transiently suppressed during acute stress to conserve energy (e.g., salt-responsive *GmRER5/6* down-regulation in leaves; Figure 5b), reflecting a growth–defense tradeoff common in annual crops [65]. Conversely, Class II paralogs (root-enriched *GmRER4a/b*; Figure 5c) prioritize stress mitigation through sustained activation, mirroring the “root-first” drought response strategy observed in other legumes [66,67,68,69]. Additionally, this dynamic modulation of leaf fatty acids, such as the heat-induced accumulation of linolenic acid [16], may complement GmRER-mediated stress responses. Intriguingly, Class I and II genes exhibited ectopic activation in non-native tissues under abiotic stress, e.g., GmREa/b and *GmRER1a* in leaves, and *GmRER5a/b* in roots (Figure 5), implying latent regulatory flexibility critical for soybean resilience.

The transcriptional polymorphism observed across northern spring soybean landraces (Figure 7c) suggests that artificial selection has leveraged *GmRER* diversity for local adaptation. For example, the parallel expression patterns of *GmRER4a* and *GmFAD3-2a* (an enzyme responsible for the synthesis of α-linolenic acid) in six landraces (Figure 7) imply co-regulated lipid-remobilization pathways. It is noteworthy that the expression profiling of the six landraces identified in this study was performed with three biological replicates per genotype. Hence, the possibility cannot be ruled out that the observed variations in gene expression may have originated from intra-cultivar variations [70,71,72,73]. For soybean breeding trials, enhancing the polyunsaturated fatty acid content (e.g., linolenic acid under high temperature) via *GmRER4a* editing could simultaneously improve thermotolerance and oil-based defense metabolites. However, the incomplete annotation of lipid-associated interactors (Figure 4a) currently limits network-level insights. Integrative multi-omics (e.g., lipidomics or ChIP-seq) studies are needed to map regulatory hierarchies of GmRERs and identify master regulators of soybean leaf oil metabolism.

## 4. Materials and Methods

### 4.1. Growth Conditions

The seeds of six landrace genotypes (L1–L6) from soybean plants that had undergone more than five consecutive generations of self-pollination were selected and surface-sterilized with 75% ethanol for 30 s followed by two rinses with RO water before cultivation. The seeds were sown individually in pots (one plant per pot), which were filled with sterilized soil and placed in a growth chamber under controlled conditions: 18 h light/6 h dark photoperiod, with day/night temperatures maintained at 25 °C/22 °C, respectively. After the plants reached full development of the first trifoliate leaf, part of each first trifoliate leaf was harvested and immediately frozen in liquid nitrogen. Three independent biological replicates per genotype (corresponding to three separate pot-grown plants) were collected and stored separately at −80 °C for subsequent RNA extraction and transcriptome analysis. It is critical to acknowledge that the use of triplicate biological replicates per genotype was limited because of the intra-cultivar variations.

### 4.2. Genome-Wide Identification of GmRER Genes

The soybean (*Glycine max* cv. Williams 82) genome (assembly Wm82.a4.v1) was retrieved from Phytozome v13 [36]. To identify RER homologs, the protein sequences of seven Arabidopsis RER genes (AtRE and AtRER1-AtRER6) were extracted from TAIR (https://www.arabidopsis.org/ (accessed on 1 April 2025)) and subjected to InterProScan (version 5.70-102) to confirm the presence of the DUF3411 (PF11891) domain [74]. These sequences were then used as queries for BLASTP searches (e-value ≤ 1 × 10^−10^) against the soybean proteome. To investigate genomic synteny relationships, the BLASTP matches were subsequently analyzed through MCScanX (with default parameters) to detect collinear chromosomal regions [75]. Gene duplication events were further characterized using the built-in gene classification algorithm in MCScanX. Candidate sequences containing the full-length DUF3411 domain were further validated via HMMER v3.3.2 using the PF11891 HMM profile (cutoff: 1 × 10^−50^) [76]. Non-redundant candidates were assigned systematic names based on phylogenetic clustering (e.g., GmRER1a, GmRER3b) following established nomenclature guidelines.

### 4.3. Molecular Evolution Analysis and Chromosomal Locations

Multiple sequence alignment of RER proteins from six species (*Arabidopsis thaliana*, *Glycine max*, *Oryza sativa*, *Gossypium hirsutum*, *Zea mays*, and *Triticum aestivum*) was performed using ClustalW in MEGA12 with the default parameters [77]. To ensure alignment integrity, gaps and ambiguous sites were treated using the complete deletion method, removing all positions containing gaps or missing data prior to downstream phylogenetic analysis. A maximum-likelihood (ML) phylogenetic tree was constructed in MEGA12. The bootstrap consensus tree inferred from 500 replicates was taken to represent the evolutionary history of the taxa analyzed, where branches corresponding to partitions reproduced in less than 50% of replicate trees were collapsed. The percentage of replicate trees in which the associated taxa were clustered together (500 replicates) was shown next to the branches. The initial tree for the heuristic search was selected by choosing the tree with the superior log-likelihood between a Neighbor-Joining (NJ) tree and a Maximum Parsimony (MP) tree. The NJ tree was generated using a matrix of pairwise distances computed using the p-distance. The MP tree had the shortest length among 10 MP tree searches, each performed with a randomly generated starting tree. The final dataset encompassed 56 amino acid sequences with 796 positions in the final dataset. Evolutionary analyses were conducted in MEGA12 utilizing up to 4 parallel computing threads. Orthology relationships were validated via bidirectional best-hit BLASTP (BBH; e-value ≤ 1 × 10^−10^) and visualized using TBtools (version 2.056) [78].

### 4.4. Gene Structure and Conserved Motif Annotation

Exon–intron structures were mapped using the Gene Structure Display Server [79]. Conserved motifs were identified via MEME Suite v5.4.1 with the parameters set to discover 10 motifs (width: 6–50 residues; any number of repetitions) [80]. Motif annotations were inferred by cross-referencing with the InterPro and SMART databases [81]. Transmembrane helices and chloroplast transit peptides (cTP) were predicted using TMHMM v2.0 and TargetP v2.0, respectively [82,83].

### 4.5. Cis-Element Profiling and Promoter Analysis

A 2 kb upstream sequence from each GmRER transcription start site (TSS) was extracted from the soybean genome. *Cis*-regulatory elements were annotated using PlantCARE with a significance cutoff of *p* < 0.01 for motif occurrence [84]. Elements were categorized into stress-responsive (e.g., MYB, ABRE), hormonal (e.g., ERE, AuxRR), and developmental (e.g., G-box, HD-Zip) modules. Heatmaps visualizing motif density (elements/kb) were generated using TBtools.

### 4.6. Protein Interaction Network Construction

Protein–protein interaction (PPI) networks were predicted using STRING v11.5 with a high-confidence interaction score ≥0.7 [85]. The networks were imported into Cytoscape v3.9.0 for modular analysis, applying the MCODE plugin to identify densely connected clusters [86]. Functional annotations for interactors were derived from the Gene Ontology (GO) database [87].

### 4.7. Expression Profiling and Data Visualization

For spatiotemporal analysis of gene expression under different conditions, normalized RNA-seq data (FPKM) were retrieved from the NCBI Sequence Read Archive under accessions PRJNA238493 (tissue atlas) [43], PRJNA246058 (salt stress) [44], and PRJNA574626 (drought/submergence) [42]. HISAT2 v2.2.1 and StringTie v2.2.0 were used for alignment and quantification, respectively [88,89]. Heatmaps were plotted using the R package pheatmap with z-score normalization (row-wise) and hierarchical clustering (Euclidean distance, complete linkage). Expression trends under stress (e.g., suppression at 4 h, recovery at 24 h) were qualitatively assessed based on hierarchical clustering patterns and the manual inspection of fold-changes.

### 4.8. RNA Extraction and Sequencing

Total RNA were extracted from each plant using the Trizol method, strictly following the manufacturer’s protocol. The RNA samples were then sent to a sequencing company for library preparation and sequencing on the NovaSeq 6000 platform. Raw reads were processed to remove adapter contamination, low-quality reads, and reads containing more than 5% ambiguous bases (N). Each sample yielded at least 5 Gb of clean data.

### 4.9. RNA-seq Data Processing, Mapping, and Differential Expression Analysis

After sequencing, raw RNA-seq reads were aligned to the cultivated soybean reference genome (Glycine max Williams 82, assembly version a4v1) using STAR (v2.7.0d) with default parameters, retaining only uniquely mapped reads for downstream analysis. Mapping efficiency for all six landraces (L1–L6) showed consistently high quality, with the raw read counts per replicate ranging from 16.2 to 19.8 million, unique mapping rates of 86.9–88.7%, and overall mapping rates exceeding 96% (complete data in Table S4). The gene expression values were filtered to retain only genes with raw counts ≥10 in all three biological replicates. Spearman correlation analysis was performed using the cor.test function in R with exact *p*-value calculation disabled for large sample sizes. Scatterplots were generated using ggplot2 (Figure S3).

Normalization and differential expression analysis were performed using DESeq2 with default parameters, including median-of-ratios normalization and dispersion estimation via a negative binomial generalized linear model. Pairwise comparisons between all six landraces were conducted using DESeq2’s Wald test, with significance defined as a raw *p*-value < 0.05. The expression values were represented as log2-transformed DESeq2-normalized counts for visualization. Boxplots were generated by using ggplot2, and statistical significance annotations (lowercase letters) were generated using the multcomp package in R. For each gene, a pairwise *p*-value matrix was constructed from all genotype comparisons, and letter groupings were assigned through the general linear hypothesis procedure when *p* < 0.05. Groups sharing the same letter indicate no significant differences.

### 4.10. RNA Secondary Structure and Protein Similarity Assessment

Full-length mRNA sequences (5′ UTR, CDS, 3′ UTR) were folded using RNAfold v2.4.18 under default thermodynamic parameters [90]. Structural similarity scores between paralogs were computed using RNAforester with the Sankoff algorithm [91]. For protein similarity assessment, three-dimensional structures were predicted using AlphaFold2 in ColabFold with default templates and multiple sequence alignments (MSAs) generated from UniRef30 (2021-09) [92,93]. Structurally aligned models were subsequently analyzed in PyMOL v2.5 to determine the root-mean-square deviation (RMSD) values as quantitative measures of structural similarity [94].

## 5. Conclusions

This study systematically characterized the GmRER gene family in soybean, which is associated with chloroplast development and plastid metabolism. We further elucidated its evolutionary trajectory, structural diversification, and functional plasticity. A total of 14 *GmRER* genes were identified and classified into six paralog groups through phylogenetic analysis. The chromosomal distribution highlighted the contributions of segmental and tandem duplications to GmRER family expansion. Architectural dissection revealed subclade-specific gene structures and conserved motifs, suggesting functional divergence. Moreover, despite divergent RNA secondary structures, the GmRER protein folds remained evolutionarily constrained, highlighting strong functional conservation overriding transcriptional plasticity. The expression profiling and promoter analysis of *GmRERs* implicated their opposing regulatory strategies for photosynthesis and energy metabolism. Remarkably, substantially different expression levels within and between paralog groups highlight potential genetic diversity within the *GmRER* gene family during soybean domestication, offering potential targets for breeding optimization. It is important to know that intra-cultivar variations may constitute a confounding factor underlying the expression divergence in our RNA-seq analysis. Collectively, our findings may not only provide critical insights into the chloroplast-localized trade-off between photosynthesis and lipid biosynthesis elucidated via GmRERs, but also offer a possibility for developing high-oil soybean cultivars.

## Figures and Tables

**Table 1:** Orthology-based identification and integrating properties of GmRERs.

Gene	Arabidopsis Gene ID	Name	Type of Duplicattion	Location (Mb)	Protein Length (aa)	Protein pI	ProteinMW (Kda)	Subcellular Localization
RE	AT2G37860	GmREa	WGD	16.276–16.281	444	4.813	47.64	Chloroplast
		GmREb	WGD	45.830–45.836	446	5.688	47.94	Chloroplast
RER1	AT5G22790	GmRER1a	WGD	35.553–35.558	385	7.5	41.37	Chloroplast
		GmRER1b	WGD	40.846–40.851	387	8.021	41.53	Chloroplast
RER3	AT3G08640	GmRER3a	WGD	38.020–38.022	321	10.196	34.04	Chloroplast
		GmRER3b	WGD	49.235–49.238	350	8.476	36.94	Chloroplast
		GmRER3c	WGD	50.736–50.739	348	9.149	36.92	Chloroplast
RER4	AT5G12470	GmRER4a	WGD	1.403–1.408	365	8.461	38.8	Chloroplast
		GmRER4b	WGD	7.082–7.087	369	9.474	39.38	Chloroplast
		GmRER4c	Dispersed	12.077–12.090	432	9.722	46.96	Chloroplast
		GmRER4d	WGD	0.697–0.702	373	8.944	39.82	Chloroplast
		GmRER4e	WGD	45.552–45.556	377	9.156	40.16	Chloroplast
RER5	AT2G40400	GmRER5	WGD	1.682–1.690	748	8.777	82.64	Plasma membrane
RER6	AT3G56140	GmRER6	WGD	37.799–37.807	749	8.44	82.63	Plasma membrane

## Data Availability

The RNA-seq data produced in our study have been submitted to the NCBI SRA database and can be found under the following accession number: PRJNA1246969. The other RNA-seq datasets analyzed in this study are publicly available in the NCBI Sequence Read Archive under the following accession numbers: PRJNA238493 (tissue-specific expression atlas) [43], PRJNA246058 (salt stress experiments) [44], and PRJNA574626 (drought and submergence experiments) [42]. The predicted protein structures generated via AlphaFold can be found at https://alphafold.com/ (accessed on 11 January 2025). The phylogenetic trees, motif annotations, and expression heatmaps have been included as figures in the manuscript.

## Supplementary Materials

The following supporting information can be downloaded at: https://www.mdpi.com/article/10.3390/plants14101516/s1, Figure S1: Predicted RNA secondary structure of the GmRER genes; Figure S2: Protein Structures of GmRERs; Figure S3. Spearman correlation analysis between biological replicates; Table S1: List of RER genes in soybean; Table S2: Sequences and Descriptions of MEME analyzed motifs in GmRERs; Table S3. Uniprot ID and annotations of GmRER interacting proteins; Table S4. Summary of mapping statistics for the six landrace RNA-seq data.

## Abbreviations

The following abbreviations are used in this manuscript:

ABA	Abscisic acid
ABRE	Abscisic acid-responsive element
CDS	Coding sequence
CRISPR	Clustered Regularly Interspaced Short Palindromic Repeats
ERE	Ethylene-responsive element
FPKM	Fragments Per Kilobase of transcript per Million mapped reads
GO	Gene Ontology
MYB	MYeloBlastosis transcription factor family
PPI	Protein–protein interaction
RER	RETICULATA-RELATED
RMSD	Root-mean-square deviation
UTR	Untranslated region
WGD	Whole-genome duplication
