# Supplementary material for: Integrative Identification of Chloroplast Metabolism-Related RETICULATA-RELATED Genes in Soybean
Source: Plants (Basel). 2025 May 19;14(10):1516. doi: 10.3390/plants14101516 (PMC12114778; doi:10.3390/plants14101516)
Supplement: Supplementary file 1 [file plants-14-01516-s001.zip › Figure S1-3.pdf]

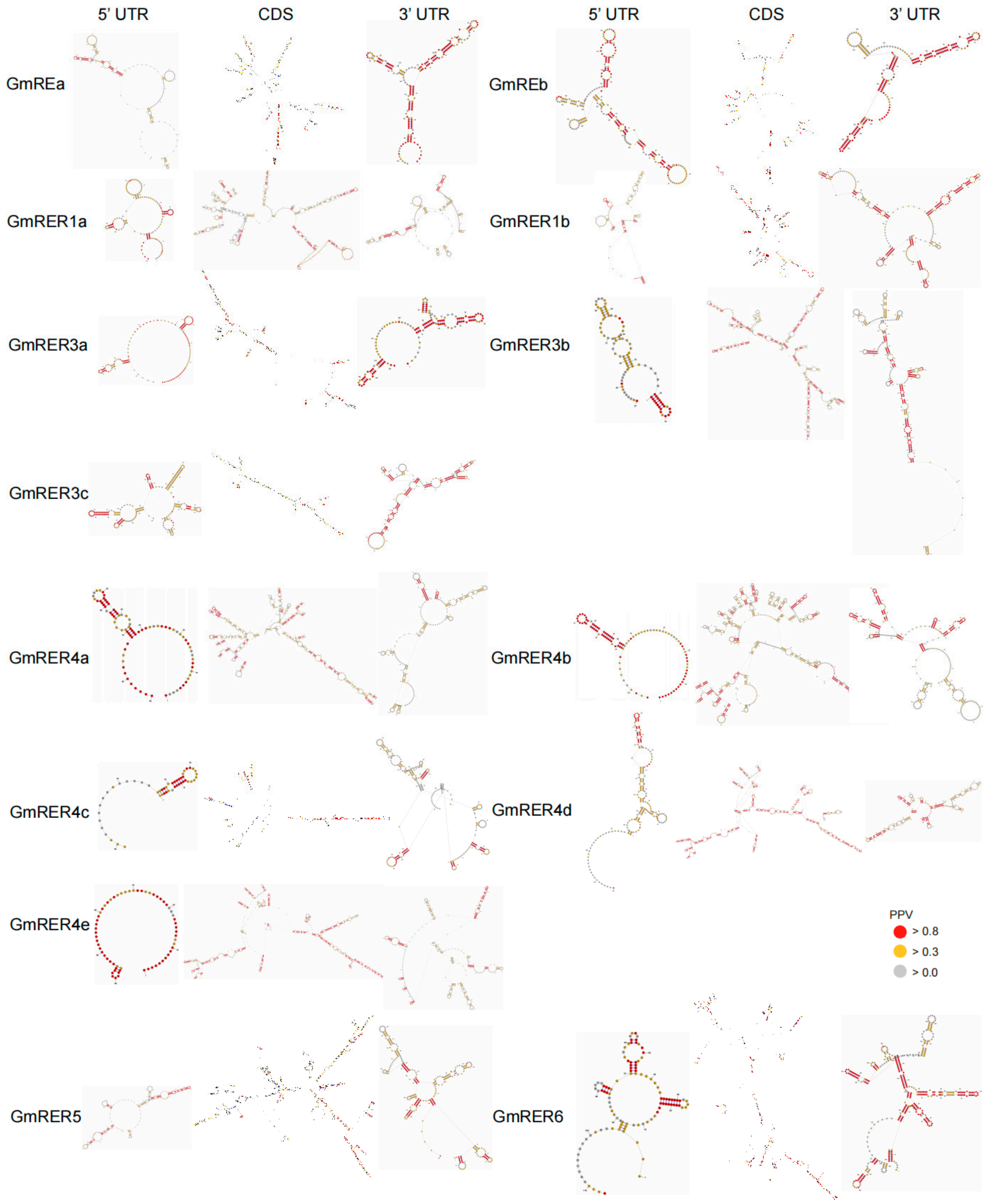

Figure S1. Predicted RNA secondary structure of the *GmRER* genes. The RNA secondary structures were computationally predicted using the Centroid algorithm implemented in RNAfold. Colors indicate base pairing probabilities: red regions denote high base pairing probability, yellow represents moderate probability, and gray corresponds to low probability. The Centroid structure represents the conformation that exhibits the minimum base-pairing distance to all other possible structures within the thermodynamic ensemble.

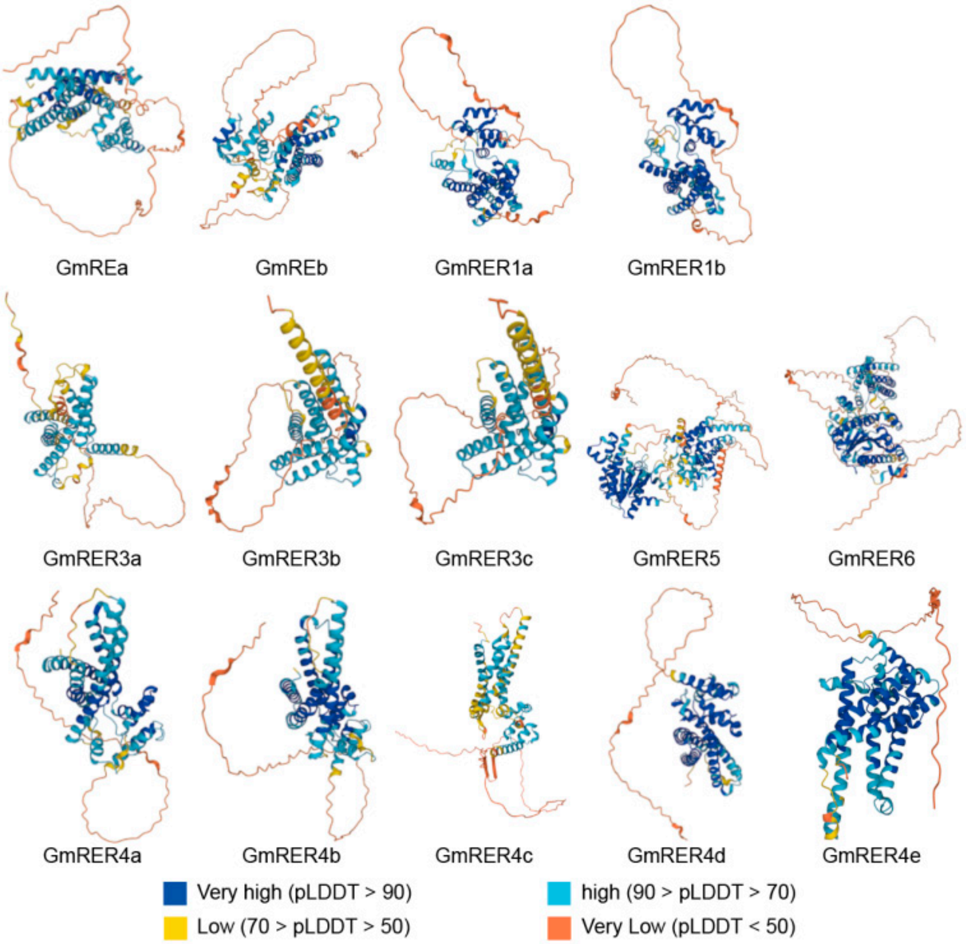

Figure S2. Protein Structures of GmRERs. The protein structures were predicted by using AlphaFold, and the confidence of the predictions was assessed by the per-residue model confidence score (ranging from 0 to 100). Higher scores indicate higher confidence in the prediction. Deep blue indicates very high confidence (pLDDT > 90), light blue indicates high confidence (90 > pLDDT > 70), yellow indicates low confidence (70 > pLDDT > 50), and orange indicates very low confidence.

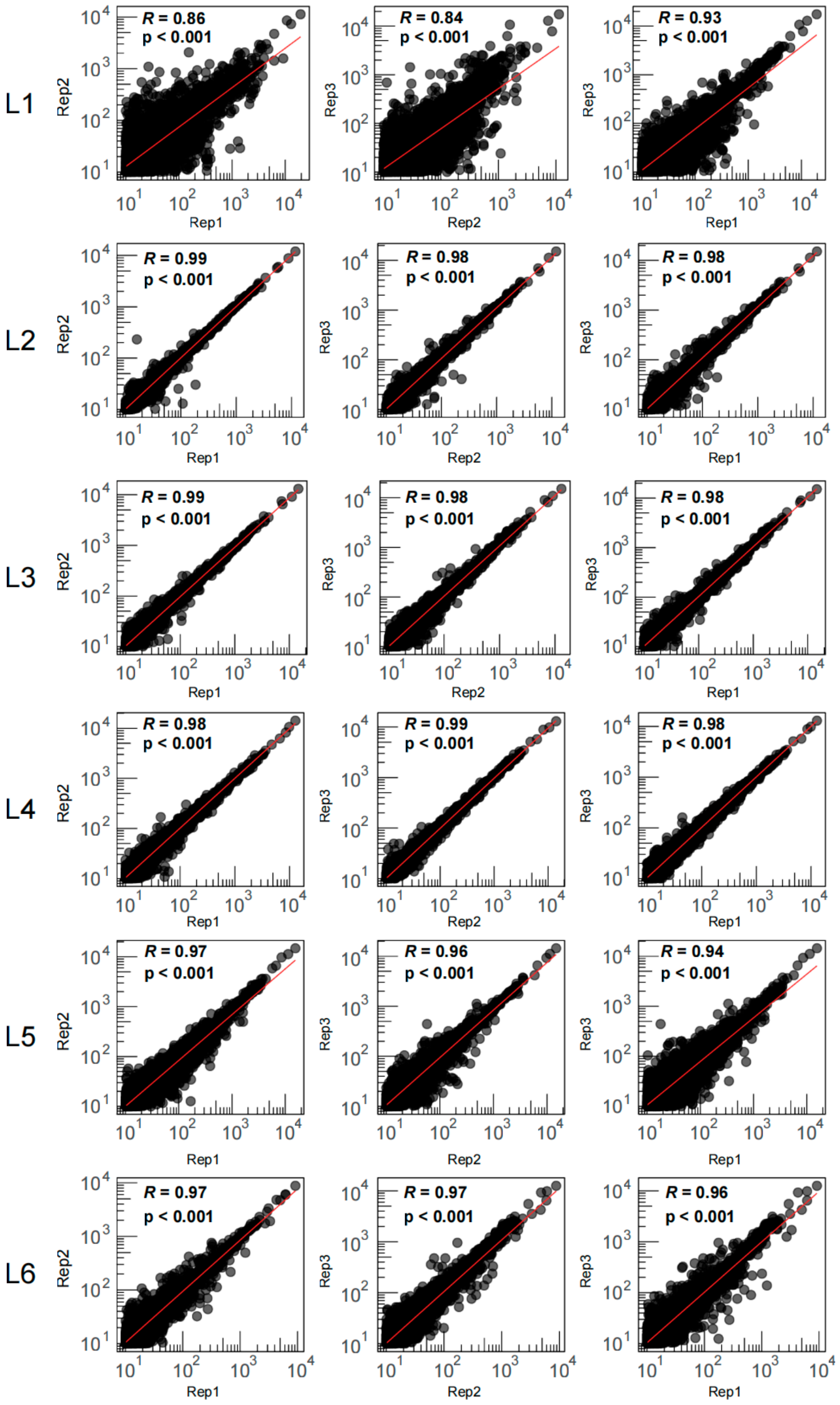

Figure S3. Spearman correlation analysis between biological replicates. Pairwise comparisons of log10-transformed expression values between three biological replicates for each experimental condition (L1-L6). Each panel shows: Rep1 vs Rep2 (left), Rep2 vs Rep3 (middle), and Rep1 vs Rep3 (right) comparisons. Points represent individual genes in all three replicates. Red lines indicate linear regression trends. Spearman's rank correlation coefficients (*R*) and corresponding *p*-values are shown in the top-left corner.
